# Editorial: New approaches to understanding vector borne diseases in domestic and wild animals

**DOI:** 10.3389/fvets.2022.1009751

**Published:** 2022-09-14

**Authors:** Elizabeth A. J. Cook, Nick Wheelhouse, Magdalena Larska, Vincent Obanda

**Affiliations:** ^1^Department of Animal and Human Health, International Livestock Research Institute, Nairobi, Kenya; ^2^School of Applied Sciences, Edinburgh Napier University, Edinburgh, United Kingdom; ^3^Department of Virology, National Veterinary Research Institute, Pulawy, Poland; ^4^Department of Veterinary Science and Laboratories, Wildlife Research and Training Institute, Kenya Wildlife Service, Nairobi, Kenya

**Keywords:** vector, emerging viruses, parasites, Orthobunyavirus, *Theileria parva*, Rift Valley fever virus, Aino virus

Arthropod vectors play a significant role in the propagation, maintenance, spread, and transmission of pathogens. Their diversity, abundance, and almost ubiquitous presence across ecosystems signify that arthropod vectors are key drivers of disease across the world, causing huge socio-economic losses due to morbidity, mortality, and loss in livestock productivity, resulting in sustained poverty and food insecurity, especially in low- and middle-income countries (LMICs).

This Research Topic invited novel contributions of the latest research regarding key advancements in the field of vector-borne animal disease. Four articles from researchers across the globe (Cameroon, Kenya, South Korea, the UK, and the USA) were accepted. The research focus of the accepted articles included the Aino virus in Korea, Corridor disease in Kenya, tick-borne diseases in Guam, and Rift Valley fever (RVF) in Cameroon.

Aino virus is a midge-transmitted Orthobunyavirus that can cause significant skeletal and neurological abnormalities in foetal ruminants exposed to the virus during gestation. Yeh and Ga investigated the Aino virus seroprevalence within wild and farmed cervids, across the Republic of Korea. They identified that both populations were exposed to the endemic virus. The authors suggest that these results highlight the need for closer monitoring of Aino virus infections in both farmed and wild cervids and that the results may help in developing future epidemiological studies of Aino virus infection.

In Kenya, Cook et al. evaluated the clinical progression of Corridor disease (CD) in naturally infected cattle. As with the more extensively characterised disease, East Coast fever (ECF), the CD is also caused by the apicomplexan protozoan parasite *Theileria parva*. However, unlike ECF, which is caused by the cattle-derived parasite, the CD is a fatal disease caused by the exposure of cattle to buffalo-derived *T. parva*. The authors identified key signs in the progression of the clinical disease that may provide indicators for early diagnosis and the basis for future effective treatment interventions.

Ticks are a significant vector for a range of infections that have a global impact on human and animal health. Weaver et al. investigated the prevalence of ticks and tick-borne diseases in dogs, cats, and wild pigs on the Pacific Island of Guam. In this wide-ranging molecular and serological study, the group identified the presence of four tick species, including a *Haemophysalis* sp. that had not been recognised on the island and a high prevalence of tick-borne pathogens, particularly in dogs. From their studies, the authors suggested that the prevalence of ticks and tick-borne pathogen exposure in dogs makes them good sentinels.

In Cameroon, Bronsvoort et al. investigated the seroprevalence of antibodies to the RVF virus (RVFV) in cattle. RVF is a zoonotic viral disease caused by the mosquito-transmitted RVFV that can cause abortion in livestock, which can act as a useful sentinel for human epidemics. While clinical RVF epidemics in East Africa are often sporadically associated with flooding events, the findings of this study suggest that in absence of apparent clinical disease and evidence of a widely circulating virus, a more stable endemic epidemiological pattern may exist in Cameroon.

The articles in this Research Topic highlight the role of vectors in endemic and emerging livestock diseases, with impacts on livestock morbidity, mortality, and production (Cook et al. and Yeh and Ga). The vector-borne disease can result in catastrophic losses to smallholder livestock keepers in LMICs, highlighting the need for better diagnostic approaches, epidemiological understanding of reservoirs, and improved control measures. Currently, available control measures, such as acaricide treatments for ticks, need to be applied frequently (at least weekly), which can be costly and limited by the development of resistance (Figure 1).

**Figure 1:**
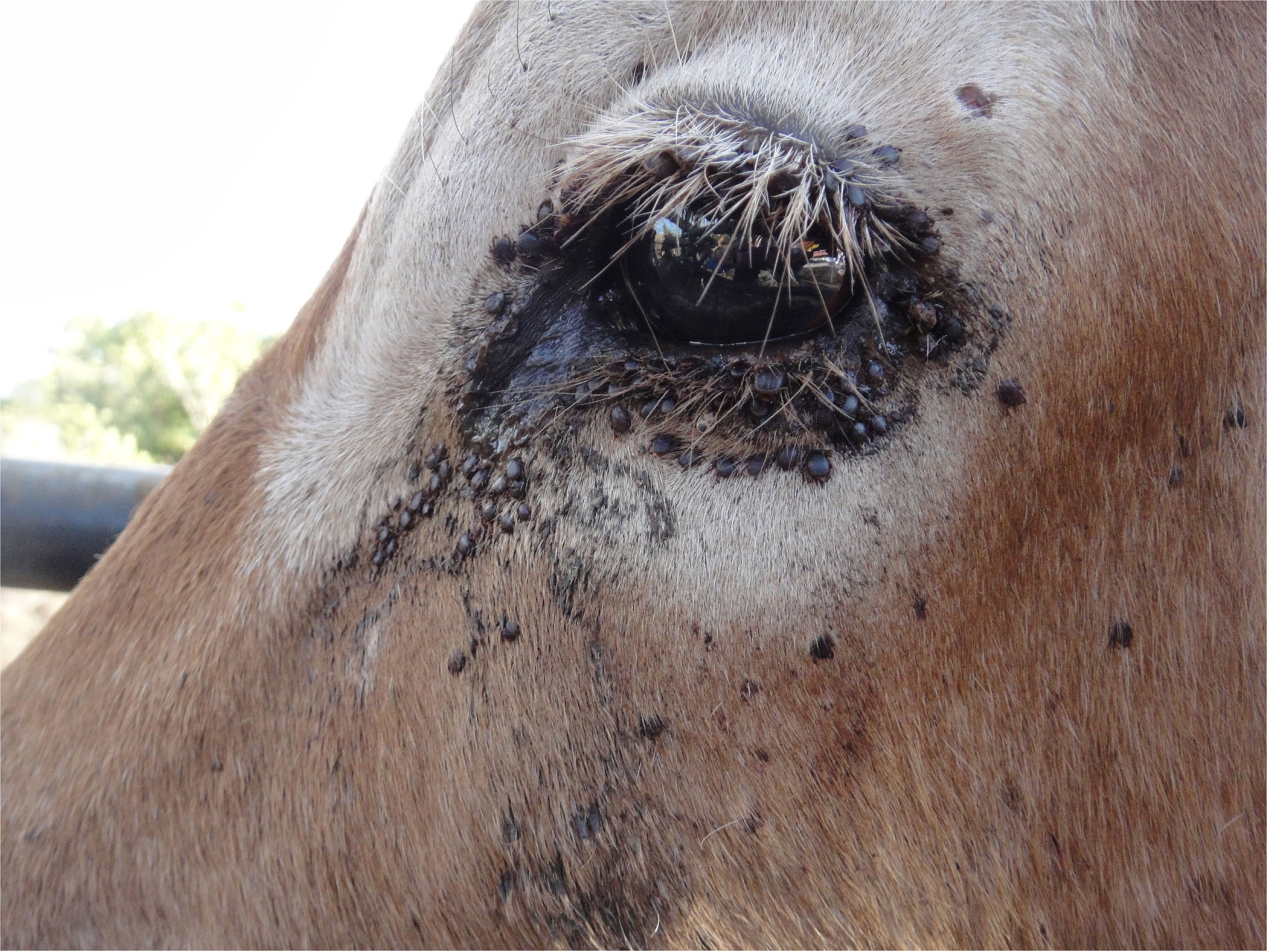
Presence of ticks around the eye of a Boran steer in Kenya that had not been sprayed with acaricide for 2 weeks (Photo credit - Elizabeth Cook).

The pathogens under study also include emerging viruses (Bronsvoort et al.) and parasites with zoonotic potential (Weaver et al.). There are two key drivers of disease emergence that may result in changes in vector distribution and patterns of disease in a reservoir and susceptible hosts, climate change, and travel/migration. The current SARS-CoV-2 pandemic and the spread of African swine fever to Asia have highlighted the potential of human and livestock pathogens to rapidly extend their geographical range. Bronsvoort et al. highlighted that transhumance may play a role in disease spread and Weaver et al. suggested that sentinels may indicate a (re)emerging threat. It is necessary to understand the current and changing distribution of vectors and their impact on animal and human health to prepare for potential emerging disease threats.

This collection of articles covers ticks, mosquitoes, and midge-borne pathogens in wildlife and livestock across three continents (Oceania, Africa, and Asia). This demonstrates the global ubiquity of vector-borne pathogens at the wildlife-livestock interface and highlights the diversity of hosts and pathogens. A One Health approach to understanding the epidemiology and clinical significance of vector-borne disease is required as evidenced by the multiple disciplines of the authors, including biologists, entomologists, immunologists, microbiologists, parasitologists, veterinarians, and virologists, of these articles. In addition, an interdisciplinary approach to controlling vector-borne diseases is required, and future work may focus on novel control methods in vectors and reservoirs.

The editors are convinced that the readers will find this Research Topic stimulating and that the information may generate novel areas for future research in vector-borne animal disease.

## Author contributions

EAJC, NW, ML, and VO assisted with the conception, preparation, and writing and editing of the manuscript. EAJC provided the image for Figure 1. All authors contributed to the article and approved the submitted version.

## Conflict of interest

The authors declare that the research was conducted in the absence of any commercial or financial relationships that could be construed as a potential conflict of interest.

## Publisher's note

All claims expressed in this article are solely those of the authors and do not necessarily represent those of their affiliated organizations, or those of the publisher, the editors and the reviewers. Any product that may be evaluated in this article, or claim that may be made by its manufacturer, is not guaranteed or endorsed by the publisher.

